# The impact of facemasks on emotion recognition, trust attribution and re-identification

**DOI:** 10.1038/s41598-021-84806-5

**Published:** 2021-03-10

**Authors:** Marco Marini, Alessandro Ansani, Fabio Paglieri, Fausto Caruana, Marco Viola

**Affiliations:** 1 Institute of Cognitive Sciences and Technologies (ISTC), Italian National Research Council (CNR), Rome, Italy; 2 Cosmic Lab, Department of Philosophy, Communication, and Performing Arts, Roma Tre University, Rome, Italy; 3 Department of Psychology, Sapienza University of Rome, Rome, Italy; 4 Institute of Neuroscience, Italian National Research Council (CNR), Parma, Italy; 5 Department of Philosophy and Education, University of Turin, Turin, Italy

**Keywords:** Psychology, Human behaviour, Emotion, Social behaviour

## Abstract

Covid-19 pandemics has fostered a pervasive use of facemasks all around the world. While they help in preventing infection, there are concerns related to the possible impact of facemasks on social communication. The present study investigates how emotion recognition, trust attribution and re-identification of faces differ when faces are seen without mask, with a standard medical facemask, and with a transparent facemask restoring visual access to the mouth region. Our results show that, in contrast to standard medical facemasks, transparent masks significantly spare the capability to recognize emotional expressions. Moreover, transparent masks spare the capability to infer trustworthiness from faces with respect to standard medical facemasks which, in turn, dampen the perceived untrustworthiness of faces. Remarkably, while transparent masks (unlike standard masks) *do not* impair emotion recognition and trust attribution, they seemingly *do impair* the subsequent re-identification of the same, unmasked, face (like standard masks). Taken together, this evidence supports a dissociation between mechanisms sustaining emotion and identity processing. This study represents a pivotal step in the much-needed analysis of face reading when the lower portion of the face is occluded by a facemask.

## Introduction

Once a rarity outside healthcare, by mid-2020 facemasks have become a pervasive feature in the everyday lives of many citizens and some local authorities made their use compulsory in many circumstances. Pervasive mask-wearing turns out to have, however, two problematic side-effects. First, by making the mouth invisible, facemasks potentially inhibit the capability to perceive a lot of social information of the utmost relevance for everyday interactions across several social contexts. To begin with, the mask can interfere with the recognition of its bearer’s emotional state. Moreover, since affective displays are also thought to affect first impressions of trustworthiness^1^, facemasks may also alter the perceived trustworthiness of unknown mask-bearers. By making emotional displays harder to interpret, facemasks may also compromise facial mimicry and behavioral synchrony which, in turn, boost social bonds, empathy and playful interactions^2–7^. The social information loss is even more dramatic in people with hearing deficits, since facemasks impair lip-reading and (to a lesser extent) sign language, which often relies on mouth movements among other things. Second, but equally important, is that—beside the recognition of emotions and trustworthiness—facemasks may jeopardize the *re-identification* of a previously observed (masked) face. Incidentally, this is the reason why criminals routinely use facemasks.

The impact of facemasks on emotion recognition, trustworthiness and face identity, however, is not necessarily of the same degree. The most prominent theory on face perception suggests that the recognition of emotional expressions and face identity are distinct perceptual processes encoded by independent psychological^8^ and neural^9,10^ mechanisms, with emotions and other social attributes heavily reliant on highly mobile facial regions, and facial identity mainly based upon invariant, static traits of the face. More specifically, as concerns *emotion recognition* and *trust attribution*, several experimental studies investigated the amount and type of social information conveyed by specific regions of the face, revealing that the mouth is pivotal in recognizing emotions, especially happiness^11–14^. Similarly, faces are judged as more trustworthy when the contrast of the mouth (and eye) regions is increased by means of experimental manipulations^15^. While this may suggest that facemasks could impair emotion recognition and trust attribution, by the time we ran this experiment almost every existing study employed explicit experimental manipulations on the mouth (and other facial regions) rather than ecological stimuli such as actual facemasks. As for *identity recognition,* in contrast, recent findings suggest that the recognition of faces is not necessarily related to internal features^16^. A recent study shows that a mix of internal and external features seem to weigh more in the recognition of both familiar and unfamiliar faces^17^. The same study shows that the mouth seems the least relevant feature. Identity recognition is known to be based on configural processing that get easily disrupted when the face percept is presented upside-down^18^ or its integrity is compromised due to other experimental manipulations^19^. How deeply the emotion-identity dissociation runs, and whether it depends upon different visual information and neural pathways, is still a matter of contention^20,21^. Only very recently, some studies on face perception in presence of surgical facemasks highlighted the possible impact of facemasks on emotion recognition, trustworthiness and identity^22–24^. However, to the best of our knowledge, no study has tested whether these processes are differently impaired by different mask types, e.g. comparing standard facemasks and masks with a transparent window that uncovers the mouth region.

On a practical note, it is worth stressing that the social costs of using facemasks should not be considered as a reason against their adoption. Rather, a deeper understanding of the mechanisms underpinning the processing of emotion, trustworthiness and identity might lead—from an applicative perspective—to the development of new methods to mitigate the loss of social information and, at the same time, to maximize socio-sanitary benefits.

In the present study we recruited a cohort of 122 participants (47 females; age = 33 ± 8) performing an on-line test to investigate to what extent (a) *emotion recognition*, (b) *trust attribution* and (c) *re-identification* are modulated by the presence of a *standard mask* occluding the entire mouth region, or a *transparent mask* restoring visual access to the mouth region. For these tasks, we used 48 different stimuli from the Karolinska Directed Emotional Faces (KDEF)^25,26^ and the Chicago Face Database (CFD)^27^ (see Experimental stimuli section). Items from KDEF were used for the emotion recognition and trust attribution tasks, while stimuli from CFD were employed for the trust attribution and re-identification tasks. Given that emotion and identity recognition rely on distinct processes, our expectation was that by restoring (to a large extent) visual access to the mouth region, transparent masks can facilitate emotion and trustworthiness judgments, but not necessarily identity re-identification.

## Results

**Figure 1:**
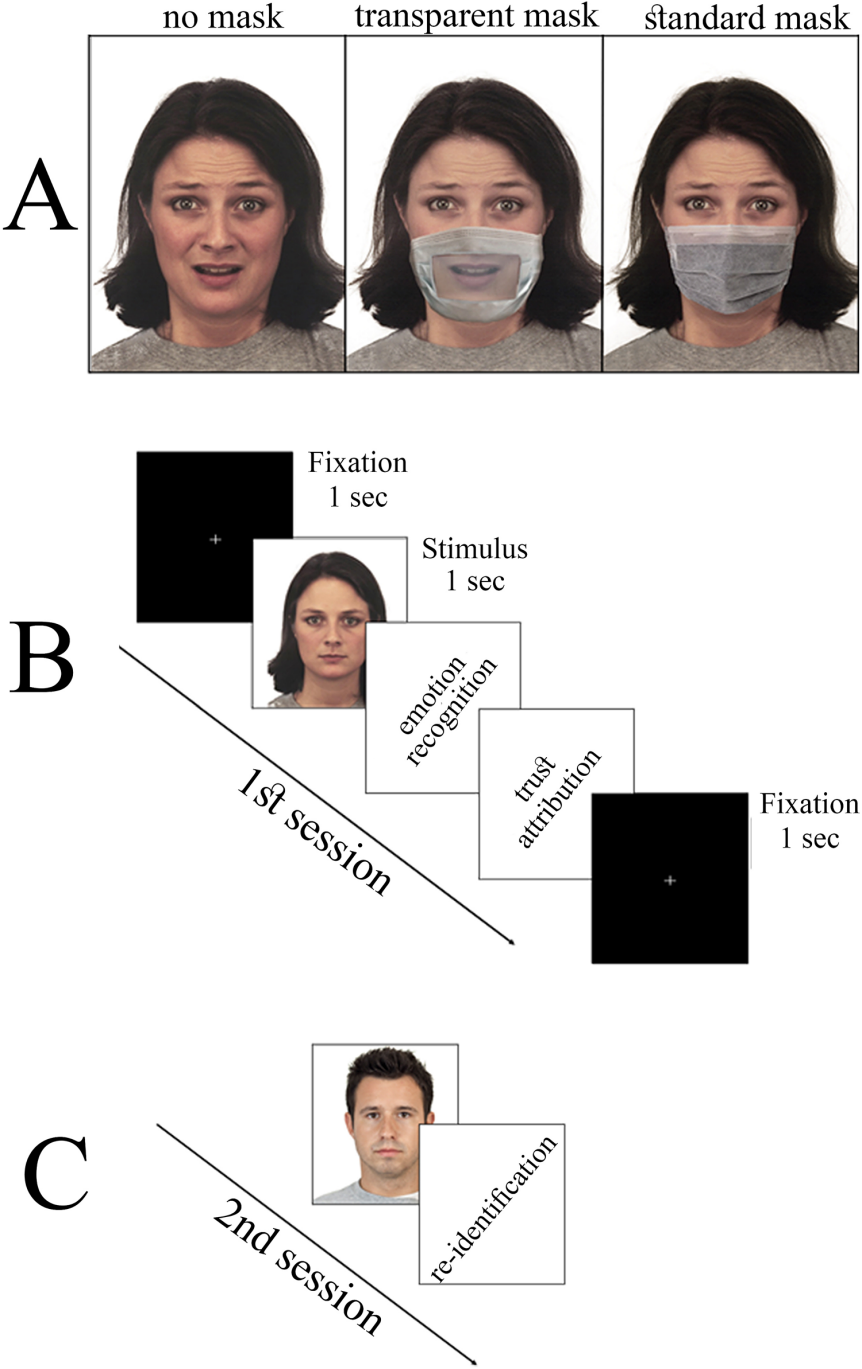
Experimental paradigm. (**A**). Stimuli consisted of faces from the Karolinska Directed Emotional Faces database (KDEF) and from a subset of the Chicago Face Database (CFD) validated for Italian subjects^26^, presented in unmasked fashion (NM, left panel), or masked with either transparent (TM, central panel) or standard (SM, right panel) medical facemasks. (**B**). In the first experimental session, participants were presented with KDEF and CFD stimuli shown in NM, TM or SM condition. After each presentation, they were required to indicate the posed emotion selecting between 4 options (Fear, Sadness, Happiness and Neutral) and, subsequently, to rate the perceived trustworthiness on a 6-point Likert scale. (**C**). In the second experimental session, participants were presented with pictures of unmasked faces from the CDF database. One third (4/12) of the presented stimuli was also presented in the first session in one of the three Conditions (SM, TM, NM). For each picture, participants were required to judge whether they have seen the face or not.

The present study was composed of two sessions. The first session was aimed at evaluating *emotion recognition* and *trust attribution* during the observation of faces posing emotional expressions (Fear, Sadness, Happiness and Neutral). Emotional expressions were posed while wearing either (1) *standard masks* occluding the entire mouth region (SM), (2) *transparent masks* restoring visual access to the mouth region (TM), or (3) *no masks* (NM; see Fig. 1A). For each picture participants were requested (a) to recognize the posed emotion, selecting between 4 options (Fear, Sadness, Happiness and Neutral), and (b) to rate the perceived trustworthiness on a 6-point Likert scale (Fig. 1B). In this experiment, we used a mixed experimental design. Due to the constraints of any online procedure, we used a between subjects design regarding the presence (and the type) of the facemask. In doing so, subjects performed only one of the three conditions (i.e., they saw only masked or unmasked faces). On the other hand, in order to assess a difference between each emotion, we implemented a within subjects manipulation by which each participant evaluated all four emotions for each of the selected faces. The second session consisted of a “*re-identification task*” aimed at investigating the impact of SM and TM in the re-identification of face identity. Pictures of unmasked faces were presented to participants, some of which already presented during the first session, and participants were required to judge whether they have seen the face or not (Fig. 1C).

### Emotion recognition in masked and unmasked faces

**Figure 2:**
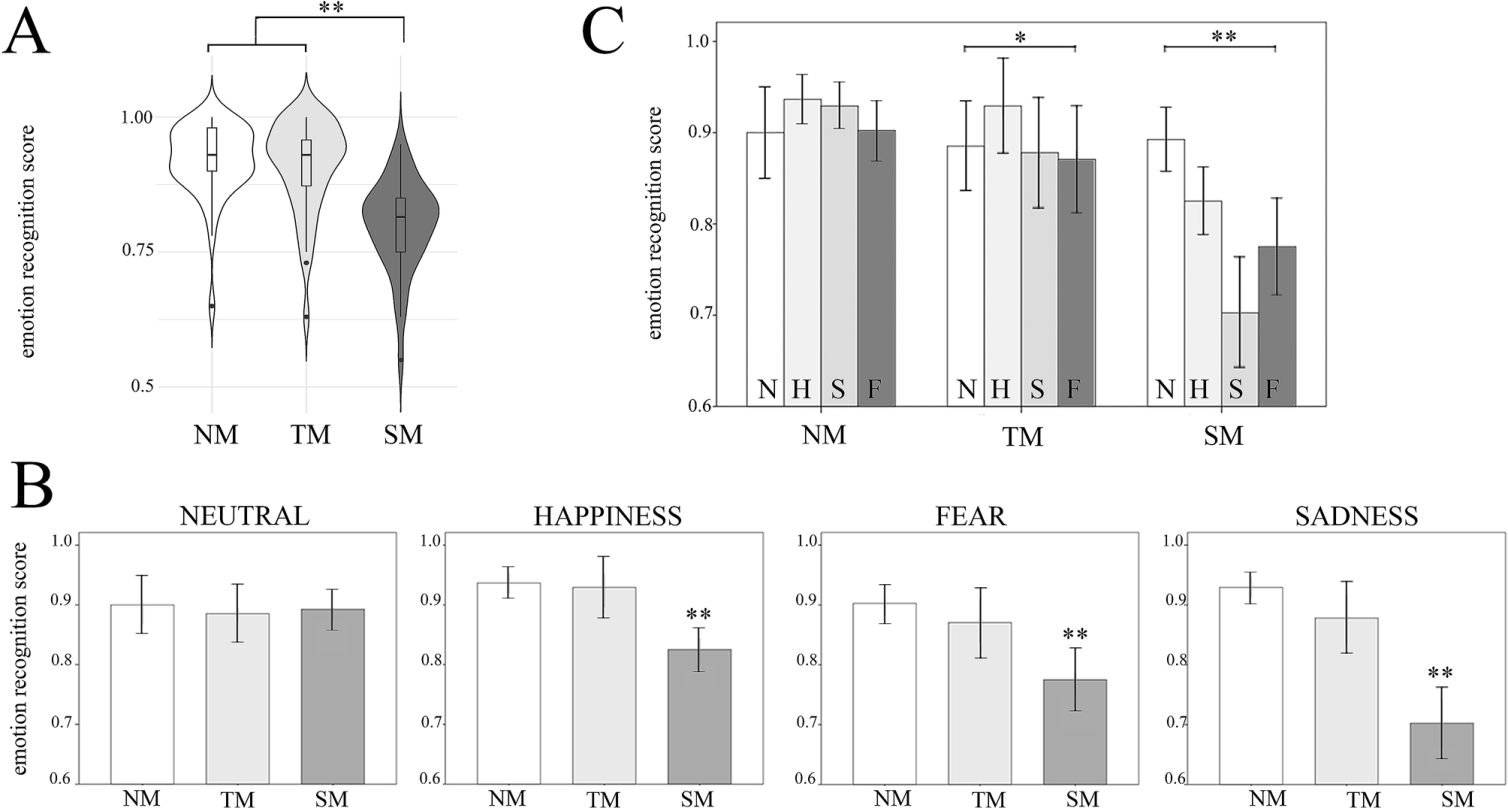
Emotion recognition task. (**A**). Violin plots depicting the accuracy of the emotion recognition task, regardless of the posed emotion. Results show a significant main effect of Condition (TM, SM, NM). Emotion recognition in SM was significantly worse than both NM and TM, while no difference was found between NM and TM. (**B**). The figure illustrates, for each emotion, the impact of the presence/type of mask. Significant effects were found for Happiness, Sadness and Fear, with a significant drop for SM with respect to both TM and NM. No effect was found for the Neutral faces. (**C**). The figure illustrates, for each condition, which emotions were more affected by the presence/type of mask. Results show a mild but significant main effect of Emotion in TM and a stronger effect in SM, but no effect in NM. See Table 1 for post-hoc results. Error bars indicate confidence intervals. Horizontal bars indicate significant results (*p < .01; ** *p* < .001). Abbreviations: N: neutral, H: happiness, S: sadness, F: fear.

Results concerning the *emotion recognition* task were based on the scores given by each participant to the Karolinska Directed Emotional Faces (KDEF)^25,26^ stimuli (n = 40), posing Happiness, Sadness, Fear or Neutral expression. We found a significant main effect of Condition (TM, SM, NM), proving that the presence/type of mask affected the ability to recognize the posed emotions, χ^2^(2, *N* = 122) = 40.53, *p* < 0.001, p_MC_ < 0.001, 99%CI [0.000, 0.001], ε^2^ = 0.335). Interestingly, post-hoc analysis (Mann–Whitney) showed that the recognition was significantly worse in SM (N = 40 Mdn = 0.81, 95%CI [0.79, 0.83]) than in both NM (N = 41 Mdn = 0.93, 95%CI [0.93, 0.93]) (*U* = 179, *p* < 0.001, *r* = 0.67) and TM (N = 41 Mdn = 0.93, 95%CI [0.93, 0.93]) (*U* = 303, *p* < 0.001, *r* = 0.54). On the contrary, no difference was found between NM and TM (*U* = 802, *p* = 0.722, *r* = 0.03), hence suggesting that the effect of TM was comparable to NM condition, and that the accuracy in SM was significantly lower than the other conditions (see Fig. 2A).

Since facemasks could have different impact on the four different Emotions (Fear, Sadness, Happiness and Neutral), we ran four Kruskal–Wallis tests to verify if emotions were significantly affected by the presence of the mask. We found a significant main effect of Condition (NM, SM, TM) for Happiness, Sadness and Fear (H: χ^2^(2, *N* = 122) = 28.69, *p* < 0.001, *ε*^*2*^ = 0.237; S: χ^2^(2, *N* = 122) = 37.52, *p* < 0.001, *ε*^*2*^ = 0.310; and F: χ^2^(2, *N* = 122) = 19.64, *p* < 0.001, *ε*^*2*^ = 0.162)). In contrast, no effect was found for the Neutral faces (N: *χ*^2^(2, N = 122) = 1.11, *p* = 0.573, *ε*^2^ = 0.009) (Fig. 2B). As concerns Happiness, Sadness and Fear, subsequent post-hoc analysis showed a significant drop for SM with respect to both TM and NM (*p* < 0.001; Mann–Whitney tests).

To further investigate which emotions were more or less affected by the presence/type of mask, we ran, for each Condition, three Friedman tests on the emotion recognition scores. Coherently with our hypotheses, the test for the NM failed to show a main effect of Emotion (F, S, H, N: *χ*^2^(3, N = 41) = 3.81, *p* = 0.282, *W* = 0.031), suggesting that participants recognized all the unmasked expressions at the same degree (Fig. 2C). In contrast, in both TM and SM, we found a significant effect of Emotion (F, S, H, N) (TM, χ^2^(3, *N* = 41) = 10.36, *p* = 0.016, *W* = 0.084 ; SM, χ^2^(3, *N* = 41) = 23.22, *p* < 0.001, *W* = 0.193)). More specifically, as concerns TM, the ability to correctly recognize emotions was significantly better preserved in the case of Happiness, compared to Neutral (*p* < 0.05), Sadness (*p* < 0.05) and Fear (*p* < 0.01) expressions. As concerns SM, in contrast, the recognition of the Neutral expression was significantly better preserved than all emotional expressions (*p* < 0.05 for H and *p* < 0.005 S and F), and that Sadness was the most affected expression (*p* < 0.001 for N and H; *p* < 0.05 for F; see Fig. 2C and Table 1 for the whole pattern and the post-hoc analyses).

**Table 1:** The table illustrates, for each condition, the results of the post-hoc analyses (Friedman test) comparing couples of posed expressions in both the “Emotion recognition task” (upper panel) and the “Trust attribution task” (lower panel).

			Neutral	Happiness	Sadness	Fear
Emotion recognition	No Mask	N	–	− 1.24	− 0.93	− 0.08
		H	1.24	–	0.36	1.67
		S	0.93	− 0.36	–	1.39
		F	0.08	− 1.67	− 1.39	–
	Transparent	N	–	− 2.3*	0.45	1.01
		H	2.3*	–	2.41*	2.72*
		S	− 0.45	− 2.41*	–	0.51
		F	− 1.01	− 2.72*	− 0.51	–
	Standard	N	–	2.37*	4.21*	2.99*
		H	− 2.37*	–	3.43*	1.69
		S	− 4.21*	− 3.43*	–	− 2.29*
		F	− 2.99*	− 1.69	2.29*	–
Trust attribution	No Mask	N	–	− 3.64*	0.79	1.78
		H	3.64*	–	3.41*	4.02*
		S	− 0.79	− 3.41*	–	2.33*
		F	− 1.78	− 4.02*	− 2.33*	–
	Transparent	N	–	− 4.01*	0.03	1.58
		H	4.01*	–	3.35*	4.13*
		S	− 0.03	− 3.35*	–	1.95*
		F	− 1.58	− 4.13*	− 1.95*	–
	Standard	N	–	− 5.15*	− 0.88	0.15
		H	5.15*	–	4.65*	4.77*
		S	0.88	− 4.65*	–	1.64
		F	− 0.15	− 4.77*	− 1.64	–

Reported values indicate the Z statistic of the Wilcoxon signed-rank test post hoc comparisons (Bonferroni corrected). Asterisks signal significant results.

The analysis of the direction of errors, performed by a chi-square test calculated by comparing for each Emotion (N, H, S, F) the actual responses with the corresponding expected values, showed a significant effect in all conditions (*p* < 0.0001). Emotions whose real values were significantly higher than the expected ones (i.e. chi-square value exceeds the average value for that emotion) were the following. In both SM and TM, Neutral expressions were mistaken for Sad expressions. In addition, in TM both negative emotions (Sadness and Fear) were mistaken with Neutral expressions. The SM, in contrast, had a different trend, with both negative emotions (Sadness and Fear) reciprocally mistaken. In addition, Happiness was frequently mistaken with Neutral (see Table 2).

**Table 2:** The table indicates the direction of errors in the emotion categorization task.

		Neutral	Happiness	Sadness	Fear
No Mask	N	369 (0.1)	17 (2.8)	11 (0.0)	19 (5.2)
	H	**2 (7.7)**	384 (0.2)	3 (6.1)	**1 (9.4)**
	S	36 (53.7)*	8 (1.0)	381 (0.1)	20 (6.6)
	F	3 (6.1)	**1 (9.4)**	15 (1.2)	370 (0.1)
Transparent	N	363 (0.0)	18 (0.6)	32 (19.6)*	29 (13.3)*
	H	9 (2.3)	381 (0.7)	**0 (14.9)**	**2 (11.2)**
	S	34 (24.4)*	8 (3.2)	360 (0.1)	22 (3.4)
	F	**4 (8.0)**	**3 (9.5)**	18 (0.6)	357 (0.2)
Standard	N	357 (4.4)	61 (43.5)*	38 (4.6)	32 (1.0)
	H	**3 (21.2)**	330 (0.3)	**4 (19.4)**	7 (14.7)
	S	29 (0.2)	7 (14.7)	281 (4.6)	51 (21.8)*
	F	11 (9.3)	**2 (23.0)**	77 (93.8) *	310 (0.3)

For each condition, columns indicate the presented emotions (n = 410 trials for NM and TM, and n = 400 trial for SM). Rows illustrate the responses given by participants, and the chi-square value calculated by comparing each cell with the corresponding expected value (χ^2^ in parentheses). Cells in bold indicate cells whose individual chi-square value exceeds the average value in that condition. Asterisks indicate the comparisons where the real value was significantly higher than the expected one.

### Trust attribution to masked and unmasked faces

The effect of masks on trustworthiness has been studied by means of two distinct analyses, targeting stimuli of the Chicago Face Database (CFD)^27^ validated for trustworthiness (n = 8), and KDEF stimuli posing emotional expressions (n = 40), respectively.

**Figure 3:**
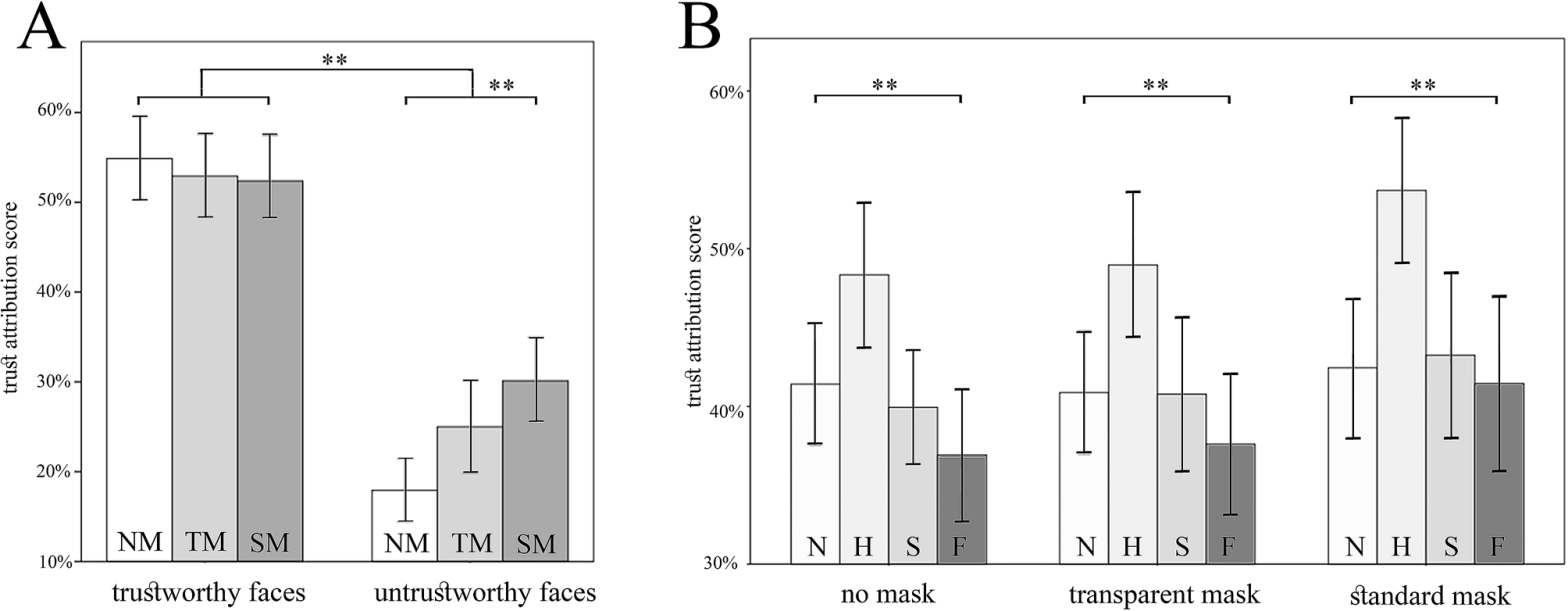
Trust attribution task. (**A**). Results for trustworthy and untrustworthy CFD faces, showing a significantly lower score for untrustworthy faces. Stimuli rated as untrustworthy shows a significant effect of Condition. See Table 1 for post-hoc results. (**B**) A main effect of Emotion was observed in each of the three conditions (NM, TM, SM) of the KDEF stimuli, with Happiness obtaining the highest scores. See Table 1 for post-hoc results. All conventions as in Fig. 2.

The first analysis investigated to what extent the ratings of trustworthiness attributed to the CFD pictures are influenced by the presence/type of mask. A Kruskal–Wallis test applied to all CFD stimuli throughout the three Conditions (NM, SM, TM) gave no effects (*χ*^2^(2, N = 122) = 3.64, *p* = 0.161, *ε*^2^ = 0.030). We then subdivided all stimuli in two sets, i.e. untrustworthy and trustworthy faces, in accord with previous results^29,30^, confirming that the untrustworthy stimuli obtained significantly lower scores (*Z* = − 9.23*, p* < 0.001*, r* = 0.836; Wilcoxon signed rank test). The same analysis applied to the two sets of stimuli showed that, while no significant effects were obtained to the faces rated as trustworthy (*χ*^2^(2, *N* = 122) = 0.52, *p* = 0.770, *ε*^2^ = 0.004), the untrustworthy faces showed a significant effect of Condition (NM, SM, TM), χ^2^(2, *N* = 122) = 13.16, *p* = 0.001, p_MC_ = 0.002, 99%CI [0.000, 0.004], ε^2^ = 0.109) (Fig. 3A). Interestingly, Mann–Whitney post-hoc analysis showed a significant increase of the trust scores assigned to the untrustworthy faces in the SM condition (Mdn = 30%, 95%CI [27.5, 30]) compared to the NM condition (Mdn = 20%, 95%CI [15, 25]) (*U* = 429, *p* < 0.001, *r* = 0.41) and, albeit not fully significant, to the TM one (Mdn = 20%, 95%CI [20, 30]) (*U* = 636, *p* = 0.057, *r* = 0.21) – indicating that untrustworthy faces are rated as “less untrustworthy” when wearing TM and, even less, when wearing SM. The same procedure applied to the KDEF stimuli gave no significant results (*χ*^*2*^(2, *N* = 122) = 2.32, *p* = 0.313, *ε*^2^ = 0.019).

The second analysis investigated the effect of different emotions on trust attribution across the three conditions (NM, SM, TM) by analyzing the trust scores obtained by KDEF stimuli. A Friedman test applied to the three Conditions separately showed a main effect of Emotion in all Conditions (NM, χ^2^(3, *N* = 41) = 16.32, *p* = 0.001, *W* = 0.133); TM, χ^2^(3, *N* = 41) = 24.46, *p* < 0.001, *W* = 0.199; SM, χ^2^(3, *N* = 40) = 45.72, *p* < 0.001, *W* = 0.381) (Fig. 3B). In particular, Wilcoxon signed-rank tests for multiple comparisons showed that, in all conditions, Happy faces obtained a higher degree of trust with respect to both negative (Fear, Sadness) and Neutral expressions (*p* < 0.001). In addition, we found that Sad expressions were scored as more trustworthy than Fearful ones in NM (*p* < 0.05), and a similar trend was also observed in the TM (*p* = 0.051; see Table 1).

### Re-identification of masked and unmasked faces

The *re-identification task* was aimed at investigating the capability to correctly re-identify unmasked faces previously observed in masked (TM, SM) or unmasked (NM) fashion. To each participant, we presented pictures of unmasked faces (n = 12), some of which (n = 4) already presented in one of the three Conditions (SM, TM, NM) during the first session. For each picture, participants were required to judge whether they have seen the face or not (Fig. 1C).

**Figure 4:**
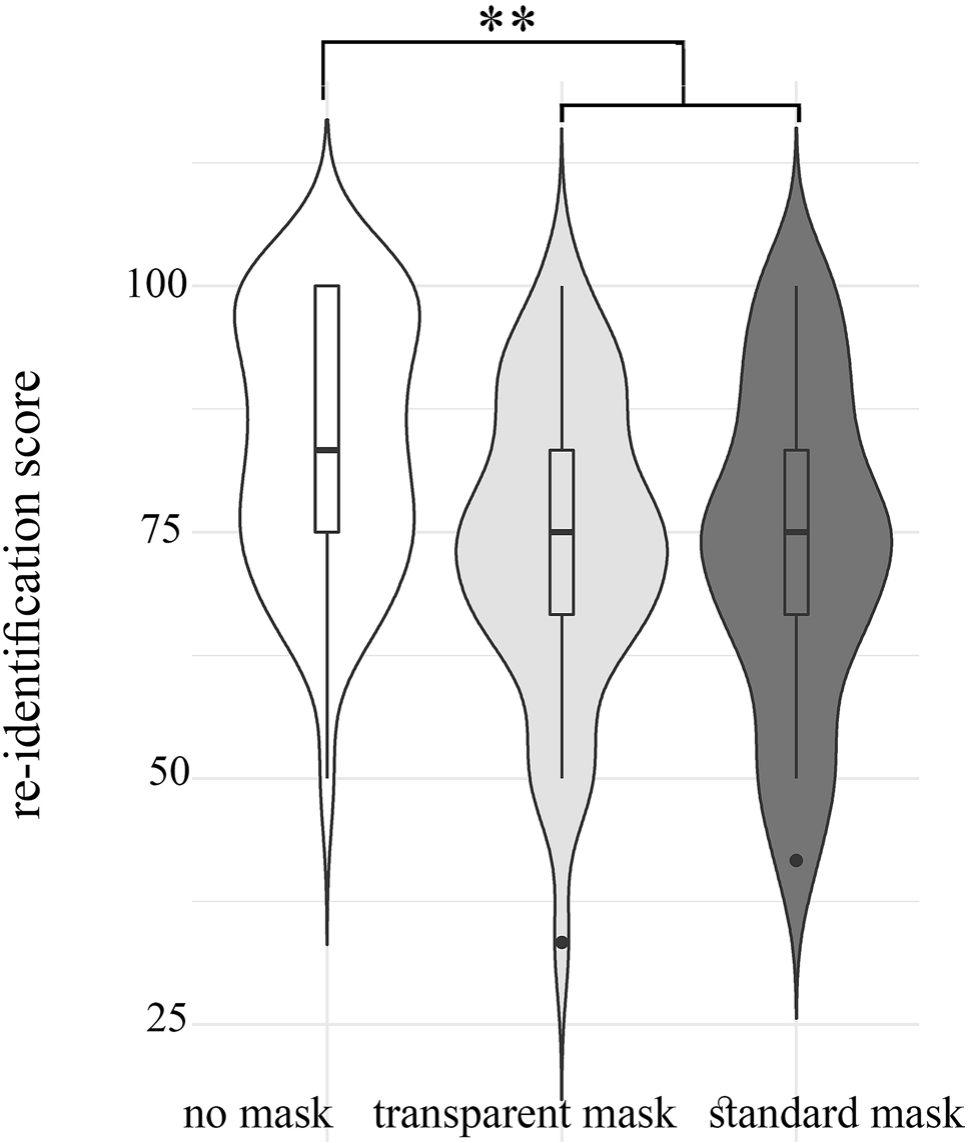
Re-identification task. The analysis of the correct re-identification of unmasked faces previously observed in masked (TM, SM) or unmasked (NM) fashion show a significant effect of Condition, indicating that the presence/type of mask affects the subsequent re-identification of that face, presented in an unmasked fashion. Boxplots within each violin represent interquartile ranges (IQRs). Black horizontal lines indicate median and black points are outliers. All conventions as in Fig. 2.

Results showed a significant effect of Condition, χ^2^(2, *N* = 122) = 11.64, *p* = 0.003, p_MC_ = 0.003, 99%CI [0.001, 0.006], ε^2^ = 0.096, CV < 20%), suggesting that the presence/type of mask affects the subsequent re-identification of that face, presented in an unmasked fashion (Fig. 4). Post-hoc analysis (Mann–Whitney test) showed that, as expected, unmasked faces were identified significantly better when previously presented without masks (NM; Mdn = 83.33%, 95%CI [83.33, 83.33]), with respect to faces previously presented with either transparent (TM; Mdn = 75%, 95%CI [66.67, 83.33]) (*U* = 520, *p* = 0.003, *r* = 0.33) or SM (Mdn = 75%, 95%CI [66.67, 75]) (*U* = 520, *p* = 0.004, *r* = 0.32). More interestingly, no statically significant differences were found between the SM and TM conditions (*U* = 812, *p* = 0.943, *r* = 0.08), indicating that both masks equally impair the subsequent re-identification of that face. Moreover, to keep into account possible random fluctuations, we firstly checked for any response bias (*e.g.*, participants who could have always responded “yes” or “no”) finding no outliers by means of a step of 1.5 × IQR. Later on, we repeated the Kruskal–Wallis test on the re-identification task considering previously seen faces and unseen faces separately. In doing so, a recodification was performed on the recall scores, so that in the case of previously seen faces, 1 point was assigned for each correctly identified face whereas 0 points were assigned for each error (i.e., “No, I’ve never seen this face”). On the contrary, for previously unseen faces, 1 point was assigned to the right answer (i.e., “No, I’ve never seen this face”) and 0 points were assigned for each error (i.e., “Yes, I’ve already seen this face”). What emerged is that the effect was significant in the case of previously seen faces (*χ*^2^(2, *N* = 122) = 6.68, *p* = 0.029, *ε*^2^ = 0.055) and not for unseen faces (*χ*^2^(2, *N* = 122) = 5.60, *p* = 0.069, *ε*^2^ = 0.046). This additional result appears to be reasonable; namely, subjects significantly failed to recognize already seen faces when they were covered by a mask; but they were unlikely to state to have seen a previously unseen face.

Lastly, all previous analyses were controlled for gender, age, residence area, and level of education. None of these factors showed significant differences (all ps = NS). Moreover, when we checked for the experiment duration in the whole sample (Mdn = 583 s. 95% C.I. [554, 608]) (Mdn = 583 s. 95% C.I. [554, 608]), we did not find any response pattern alteration (outliers)—also when we took into account the features of the stimuli (i.e., trustworthy/untrustworthy; unseen/seen faces), proving that our manipulations did not affect the response strategies.

## Discussion

In the present study we tested to what extent observing an individual wearing a standard (SM) or a transparent facemask (TM), rather than no mask (NM), alters emotion recognition and trust attribution, as well as incidental episodic memory of previously observed face. We found that, as expected, standard masks (a) interfere with emotion recognition and trust attribution, and (b) make it harder to re-identify an already encountered face. More interestingly, we found that transparent masks (c) exert minimal to no effect on emotion recognition and trust attribution, but (d) they complicate re-identification as much as standard masks. In the following sub-sections we briefly discuss each of these aspects along with some possible implications.

### Emotion recognition and facemasks

Observing emotional expressions in individuals wearing different types of masks alters the observer’s processing of emotion in a different manner. In particular, while standard masks impair the detection of facial displays, transparent masks—which restore visual access to the mouth region—have virtually no effects on emotion recognition, leading to results that are comparable to those obtained when the face is fully visible. Of note, this effect was particularly strong in the case of the three emotional expressions, but virtually absent in the case of the neutral expression, which was indeed correctly recognized in all conditions.

The evidence that transparent masks do not impair the recognition of emotions suggests that emotional displays are largely detected on the basis of specific individual details—and the mouth in particular—rather than on a holistic processing of the whole face. This hypothesis is in line with huge amount of data highlighting the role of the mouth region in the recognition of many emotional expressions, and in particular happiness^13,28–30^, thus suggesting that transparent masks provide a workable alternative to standard masks to face the Covid-19 emergency and, at the same time, to allow individuals to share emotions and to convey face-mediated social intentions and non-verbal communication in a standard fashion.

As concerns the standard masks, we observed an overall impairment in emotion recognition, as reported in a previous study^22^. However, the obstruction of information from the mouth region does not have the same impact on all four emotions. Not only the standard mask spares the detection of the neutral expression, it also has a particular impact on the recognition of sadness. Indeed, the mean accuracy drop for sadness recognition (from 93% in NM to 70% in SM) is more than twice that of happiness recognition (from 94% in NM to 83% in SM). This is particularly intriguing, because the mouth is known to be particularly relevant for detecting happiness over other negative emotions^31^. However, this may be due to a ceiling effect in the NM condition. Moreover, that happiness is easier to recognize than other emotions is shown both by classical literature on emotion recognition in normal conditions^32–34^ and by recent literature that makes it harder by adding visual noise^11,35^. Hence, the addition of a (standard) mask might simply have “unmasked” the higher difficulty of recognizing sadness as compared to happiness. It has been speculated that this higher performance might depend on the fact that, within our stimuli, happiness is the only positive emotion, among the classical six basic emotions^36^*.* In fact, Table 2 reveals that in standard mask condition sad faces were often mistaken for fearful faces, and vice-versa. Moreover, while in some cases participants confused either positive (happy) or negative (fearful or sad) expressions with neutral ones, they almost never reverse the valence polarity of expressions (i.e., they seldom confused positive and negative faces; see also Fig. 5 in Carbon’s study^22^). It is thus likely that, while full facial information facilitates the recognition of a specific emotion category, less facial information (from the upper part) is sufficient to correctly assess the valence of some facial expression, as if valence is redundantly expressed by several facial features. Indeed, the view that the recognition of valence from facial expression (that Russell dubbed “minimal universality”^36^) is more fundamental than that of specific emotion categories seems supported both on the ground of developmental psychology^37^, and by recent cross-cultural studies^38^. Further research may directly address the hypothesis that *minimal universality* in emotion recognition “pierces the mask”.

Another interesting finding is that, consistently with a previous study^22^, masks make no difference for identifying that a face is neutral with respect to emotional. While people do seem to treat “neutral face” as a proper category^39^, the emotional meaning of neutral faces may be influenced by the context^40^. However, previous literature strongly suggests that the emotional neutrality of a face can be easily decoded by the eyes^41,42^.On a more practical side, the data suggest that transparent masks almost entirely avoid the “emotional screening” effect of standard masks. Indeed, the accuracy of emotion recognition of faces wearing transparent masks is almost comparable to that obtained with unmasked faces, and significantly better than that obtained with faces wearing standard masks, for all emotions.

### Trustworthiness and facemasks

The effect of masks on trustworthiness has been studied by means of two distinct analyses. First, we established to what extent the ratings of trustworthiness attributed to the CFD pictures—where scores for trustworthiness of unmasked faces are validated—are influenced by the presence/type of mask. While the perceived trustworthiness of faces validated as “trustworthy” in the CFD remains stable between NM, TM and SM conditions, things go differently once we consider those faces that, according to the CFD, are “untrustworthy”. In other words, the low trust judgments on untrustworthy faces in the “no mask” condition are consistent with those of the CFD, but their scores are less negative in faces wearing transparent masks, and even less so in faces wearing standard masks—albeit they never reach the score of trustworthy faces. In a sense, it looks like “untrustworthiness” gets screened by masks.

The second analysis, aimed at exploring the link between emotions on trustworthiness, was performed on the results obtained from the presentation of the KDEF stimuli where, however, validated scores for trustworthiness are not available. Here, we found that the posed emotional expression significantly affects the degree of attributed trust, with sad expressions rated as more trustworthy than fearful ones in both NM and TM conditions, but similar in the SM condition. Such assimilation of transparent masks and unmasked faces is in line with our previous observation that these conditions are perceived as very similar, with respect to the standard masks (see above). The fact that sadness has lower impact on trust than other emotions with negative valence, e.g. fear and anger, is already documented in the literature^43,44^: a possible explanation considers that sadness is not necessarily directed at someone, whereas both fear and anger typically have a specific target. If that target is the trustor, then this gives a good reason not to trust that trustee—it would be risky to rely on someone who is scared of us or mad at us. But even if the emotional target is perceived as external to the trust relationship, it may elicit the assumption that the trustee’s attention is directed elsewhere, which in turn makes him/her not particularly trustworthy. These reasons against trust are absent with respect to sad trustees. Moreover, a sad face is often perceived as particularly vulnerable. In turn, a vulnerable/weak person might be seen as unlikely to defect or backstab the observer, and thus, more deserving of trust.

Happy faces, in contrast, lead to higher degrees of assigned trust over both neutral and negative (sadness and fear) expressions in all conditions, that is, regardless of the presence/type of mask. The high scores in trustworthiness obtained by happy faces can be easily explained by the intrinsic affiliative and approach-oriented nature of smile and laughter^3,45–48^ and by the evidence that observing such expressions induces automatic facial mimicry and emotional contagion^49,50^. Note also that observing happy expressions boosts the activation of the same emotional regions controlling the production of the same positive expression, namely a “mirror mechanism” for laughter and smiling^45,49,51^. The high scores obtained by happy expressions can be also explained from a dimensional standpoint and, in particular, from the hypothesis that trust attribution depends on a combination of valence and dominance^52^. Following this view, valence and dominance are established through a process of overgeneralization of their similarity with emotional expressions and with cues that signal physical strength, respectively^53^. In a slogan: the more a face expresses the willing to harm and physical strength, the lesser the trust we attribute to her bearer. Indeed, the trust scores assigned to the emotional and neutral facial stimuli drawn from the KDEF suggest a positive correlation between valence and trustworthiness, with faces expressing happiness rated as more trustworthy than those expressing negative emotions (fear or sadness) or no emotion. However, it is likely that valence alone is not enough: indeed, while sad and fearful faces both express negative emotions, the former are judged as slightly less reliable than the latter in both unmasked faces and transparent masks. This trend vanishes in the case of standard masks, possibly because participants in that condition had more trouble distinguishing between sad and fearful faces.

If trust is based on both valence and dominance, which dimension drives this screen-off? Tentative as it may be, the evidence about emotion recognition is at least suggestive that valence perception is not screened by facemasks. Moreover, the trust judgments of emotional faces discussed above reveal a same pattern of positive correlation between valence and trustworthiness across all three conditions. It is then reasonable to assume that masks screen untrustworthiness by partially obstructing cues relevant for dominance estimation. These findings are in line with a positive correlation between perceived dominance and facial width-to-height ratio (fWHR)^54^. Since the width of the face is measured on the basis of the distance between cheekbones, that are partially covered by the masks, it is possible that the untrustworthiness-screening effect of masks are mediated by the obstruction of the zygomaticus region, which yields dominance-related cues.

Intriguingly, happy faces turned out to be perceived as significantly more trustworthy in the standard mask than in either no mask or transparent mask condition. Prima facie, this seems at odds with the fact that happy faces are less often recognized as such when covered by a standard mask. While we cannot formulate any definitive explanation for this puzzling finding, three observations are in order. First, we know that some affective properties of happy faces are perceived subconsciously^55^, and that subconscious perception is sufficient to elicit emotional contagion^56^. As such, it is possible that the positivity of these faces was unavailable during a conscious categorization task, but still managed to elicit a contagion subconsciously scaffolding trustworthiness judgment. Second, note that the higher trustworthiness observed in the standard mask condition is not limited to the case of happy faces, but is pervasive in each emotion condition. This seems in line with recent findings suggesting that masked faces are perceived on average as more trustworthy than unmasked faces^23^. We speculate that, as the study was conducted during the pandemic, facemasks are taken as a proxy of social compliance and caring. This effect does not occur for transparent facemasks, possibly because they are not perceived as a medical tool to contrast the pandemic. Third, facemasks may alter the estimated distance between cheekbones, possibly inflating the perceived fWHR in our stimuli. As mentioned above, a larger fWHR may lead to higher dominance scores; and higher dominance usually implies higher trustworthiness when paired with positive valence, as in the case of happy faces.

### Re-identification and facemasks

The analysis performed on the re-identification task showed a significant drop in the accuracy when faces were first seen with a mask, as compared to when they were first seen without masks. As expected, individuals previously presented without masks were re-identified better than unmasked individuals previously presented with a mask. This is likely explained by the fact that encoding and retrieval were matched in the unmasked but not in the masked conditions. In contrast, more interesting is the lack of difference between the performances obtained with transparent and standard masks. Indeed, while the translucent window of transparent masks allows to prevent the impairment in *emotion* recognition characterizing standard masks, and only slightly influences *untrustworthiness* judgments, when it comes to *identity* recognition transparent masks are as problematic as standard ones,—suggesting that the last task relies on a different process. How can such dissociation be explained?

Previous studies demonstrate that the recognition of unfamiliar faces (such as those tested in the present study) largely depends on the visibility of external (as opposed to internal) features of the face. However, a recent study verified that the recognition of both familiar and unfamiliar faces is similarly impaired when standard masks are worn. That very same study urged the development of “transparent face coverings that can reduce the spread of disease, while still allowing the identification of the individual underneath”^24^. Based on our results, we predict that transparent masks like those we employed in this study (i.e. that only uncover the mouth region) will not. Indeed, since the mouth size has been shown to be the least relevant feature for identifying both familiar and unfamiliar faces^17^, and given that transparent masks only uncover the mouth region, while the external features of the face (e.g. the chin) remain covered, this may explain why they fail to mitigate the accuracy drop observed in the case of standard masks. It is important to bear in mind that what we are referring to constitutes a peculiar kind of re-identification, namely the incidental recognition of a previously observed, unfamiliar, face. This task should not be confused with other types of face identity-processing tasks, such as those related to visual memorization (e.g. the explicit recall to memory of a face), or to semantic processing (e.g. the explicit recognition of an individual’s name and identity). Indeed, participants were never told to memorize faces nor informed about the re-identification task that followed in the second session. In addition, all faces were unfamiliar. We opted for this methodology in the light of a more ecological design as the incidental re-identification is a process that is very similar to what happens in our daily social contacts. Nonetheless, it would be interesting in future studies to investigate the impact of the facemask on intentionally learned or familiar faces.

Barring future studies showing that standard and transparent facemasks exert a different impact on familiar faces, this dissociation between emotion and re-identification seems is in line with dual-route models of face perception positing that facial identity and emotional expressions are processed by separate cognitive mechanisms, triggered by distinct visual features^8,20,57^. It is widely accepted that, in normal conditions and in healthy observers, the process of identity recognition relies on the processing of the whole face rather than focusing on individual parts^19,20^. In contrast, emotion recognition is largely based on specific information from the mouth, or the eye, region, depending on which emotion is expressed.

Neuroscientific dual-route models of face perception suggest that emotion and identity recognition are processed by two different sectors of the temporal cortex^10,57^. Emotion recognition, relying on the identification of the changeable, and dynamic, aspects of the face, is processed in the “dorsal stream” for faces encompassing the visual motion area MT and STS areas. Face identity, in contrast, mainly relies on those aspects of the face structure that are invariant across changes (static), and is processed in the “ventral stream” for faces in the inferotemporal region^58,59^. On the basis of this perspective, we speculate that, during the presentation of the stimuli, the dorsal stream was minimally affected by the transparent mask, being the mouth fully visible. In contrast, the reduced capability of re-identify previously observed (masked) faces is telling of a more dramatic impairment of the ventral stream. The reduced functioning of the ventral stream can be accounted for by two alternative explanations. First, the capability to recognize the actor’s identity could depend on a holistic processing, which is compromised by both types of masks. While this interpretation is in line with previous hypotheses on identity recognition, one could expect that the disruption, via masking, of the holistic processing of the face should lead to a much more pronounced reduction in accuracy than the one observed in our study. An alternative, and more tempting, interpretation is that the capability to re-identify the actor relies on different types of information, not limited to the mouth/eyes regions, but also relying on cues of the lower half of the face, such as contrast reduction of the jaw and cheeks, small freckles and wrinkles, which are screened by both types of masks. To disentangle between these alternative hypotheses, further studies may compare the effect of semi-transparent vs. fully transparent masks, to investigate whether the latter are able to recover not only the capability to recognize emotions and trustworthiness, but also to better re-identify previously observed (masked) individuals.

Given the dissociation between dynamic and static features encoded by the dorsal and ventral streams respectively, one could argue that such a model cannot account for our results, being all our stimuli static. However, despite the dorsal stream for faces is indeed typically triggered by dynamic facial expressions, Furl and colleagues^58^ demonstrated that the presentation of static emotional expressions—as the ones used in our study—activated the same STS sectors typically activated by dynamic expressions, hypothesizing that static emotional expressions determine an “implied motion”, hence activating the same neuronal population encoding dynamic expressions.

### Implications

To the best of our knowledge, this study represents the first systematic enquiry concerning social readouts from faces with standard and transparent masks. Many more analyses will be needed to get a full grasp over the complex, often context-mediated, interaction between various types of masks and social information based on face perception. The present study could be fruitfully complemented by further within subject designs. Moreover, while for the sake of simplicity we have treated masks only as if they *subtract* social information by obstructing the face, it is likely that they also *add* social information of some sort. Fischer and colleagues^29^ demonstrate that the emotional meaning ascribed to women’s faces covered by a digital manipulation slightly differ from that of the same faces covered by a Niqab (a traditional Muslim veil). More closely to the object investigated here, i.e. the medical facemask, social sciences such as anthropology^60^ and semiotics^61^ offer precious insights about how its meaning may change across cultures and across times. Nevertheless, we think that some tentative implications may be legitimately drawn from our data.

First, we have seen that standard, but not transparent, masks compromise the capability to recognize the emotion (albeit probably not the valence) on the basis of facial cues. Being able to see one’s facial movements is not only useful for the sake of knowing mental states. As mentioned above, emotional decoding is likely to involve facial mimicry, which, beside its role in emotion recognition, is also thought to play a role in fostering empathy^2^–^7^. These expectations seem supported by a study conducted in Hong Kong after the SARS pandemic^62^, reporting that primary care doctors visiting patients with a medical facemask were perceived on average as *less* emphatic, especially when subjects have been patients of the same doctor for a long time. It is thus safe to assume that the possible benefits of transparent masks extend beyond enabling verbal communication with sign language, which originally inspired their design, by also favoring empathy mediated by facial mimicry. Consequently, as the social impairments brought about by facemasks partially explains why some people refuse to employ them, by partially re-enabling social communication transparent masks could mitigate the skepticism toward wearing them. Moreover, as it has been shown that empathy is pivotal in promoting compliant behaviors toward physical distancing and mask wearing^63^, by restoring the emotional display that scaffolds empathy, transparent facemasks may indirectly promote the diffusion of mask wearing itself.

However, we should refrain from the simplistic conclusion that transparent masks are *always* preferable to standard ones. Recall that facial first impressions profoundly affect observers’ behavior, often by perpetrating prejudices^64,65^. For instance, it has been recently shown that, during the triages aimed at establishing the severity and hence the priority of patients in the emergency unit of a hospital, the perceived untrustworthiness of faces predicted less severe categorization^66^. As this outcome is likely to embed some inequalities, based on our findings that masks reduce perceived *un*trustworthiness, it would be interesting to speculate whether masked patients would have received a fairer treatment.

A final implication is that masked faces are harder to recognize, even if their mouth region is observable. Trivial as it may seem, further investigating this matter will prove paramount in a context such as forensic. Indeed, as face is the more visible hallmark of personal identity, it is not by chance that in many countries the law forbids to cover the face without necessity in public spaces.

## Materials and methods

### Participants

The experiment was an on-line test (see below) carried out on 122 Italian native speakers (47 females; age = 33 ± 8), recruited by means of different social media platforms. Before the experiment, participants provided some basic demographic information (available upon request to the corresponding author), read the main instructions, and provided an informed consent. By accessing a single un-reusable link, each participant could run the experiment directly from home on their laptops, smartphones, or tablets. An anti-ballot box stuffing was employed in order to avoid multiple participations from the same device. Informed consents were requested before the experiment started and the whole procedure was approved by the Institutional Review Board (IRB) of Sapienza University of Rome (ID 0001261 - 31.07.2020). All methods were carried out in accordance with relevant guidelines and regulations.

### Experimental procedure

The experiment consisted of an on-line test (Qualtrics.com) composed by two distinct sessions. The first session (“*emotion recognition and trust attribution”*) was aimed at evaluating the impact of SM and TM on emotion and trust attribution. The second session (“*re-identification task*”) was aimed at investigating the impact of SM and TM in the capability to re-identify face identity. The entire study lasted 10 ± 4 min. Response times for each task and condition gave no significant results and were discarded from further analyses (Emotion Recognition: Mdn = 1.71 s. 95% C.I. [1.63, 1.81]; Trust Attribution: Mdn = 1.61 s. 95% C.I. [1.52, 1.70]; Recall: Mdn = 2.11 s. 95% C.I. [1.92, 2.23] ).

- *Emotion recognition and trust attribution*
  During the first session participants were asked to observe a randomized series of 48 faces clustered in 4 blocks, presented under one of the three following conditions: (a) unmasked faces (NM); (b) faces wearing a SM occluding the mouth region and (3) faces wearing a TM restoring the mouth region (see below for details). Each trial started with a 1 s fixation cross, followed by a 1 s presentation of a face presented in NM, SM or TM conditions. Regardless of the presence/type of mask, all faces posed one of the following expressions: fearful (F), sad (S), happy (H) or neutral (N). At the end of the stimulus presentation, two questions appeared on the screen. The first question (for *emotion recognition*) asked participants to answer “which emotion was expressed by the face” and participants were requested to identify the posed expression among four different options (F, S, H, N). In order to avoid any “parachute effect”, namely that undecided subjects selected the only answer implying no emotion, we characterized this option by calling it “neutral expression” instead of “none” or “no emotion. Subsequently, in a different screen, a second question (for *trust attribution*) asked “to what extent would you trust this person?”, and participants were asked to rate the perceived trustworthiness on a 6-point Likert scale. After the second answer was given, a new trial began. For each participant, the session consisted of 48 trials, and only one of the three conditions (NM, TM, SM) was randomly assigned to each participant (Fig. 1A). Moreover, to avoid any selection bias, the IT platform randomly assigned each subject to one of the three conditions in a balanced manner. This procedure made also sure that each condition was submitted to the same number of participants (NM = 41; TM = 41; SM = 40). The order of the blocks was fixed regardless of the condition, while the presentation order of the stimuli within each block was randomized to avoid any primacy or recency effects (see Table 3). This design ensured the lowest possibility of seeing the same face twice in a row. Each block was balanced for gender and facial expressions. Lastly, through a chi square test, we also verified that conditions did not differ for socio-demographics information (gender, age, residence area, and level of education; all ps = NS).
- *Re-identification task*
  The second session was aimed at evaluating the capability to recognize an unmasked face previously presented in NM, TM or SM condition. All faces in this session consisted of neutral expressions (from the Chicago Face Database; see below), and were displayed without masks, regardless of the condition the participant was assigned to in the “*emotion recognition and trust attribution*” session. For each participant, 12 faces were shown, 4 of which were already presented in the previous session, in NM, SM or TM condition. Previously presented faces were alternated, in a random order, with 8 brand new faces. The unmasked face, either previously presented or a new one, was shown on the monitor screen and participants were requested to answer whether or not they had already seen the face in the first session. To minimize both priming and recency effects, the items used in the re-identification task were always shown in the middle blocks (2 and 3) of the first session.

**Table 3:** Experimental procedure: blocks order and randomization. K = KDEF, C = CFD; f = female, m = male; N = Neutral, H = Happy, S = Sad, F = Fear, T = Trustworthy face, U = Untrustworthy face. Numbers indicate the faces’ identity.

	St. 1	St. 2	St. 3	St. 4	St. 5	St. 6	St. 7	St. 8	St. 9	St. 10	St. 11	St. 12	St. 13	St. 14
Block 1	Kf1N	Kf2H	Kf3S	Kf4F	Kf5N	Km1N	Km2H	Km3S	Km4F	Km5H				
Block 2	Kf1H	Kf2S	Kf3F	Kf4N	Kf5H	Km1H	Km2S	Km3F	Km4N	Km5S	Cm1T	Cm2U	Cf1T	Cf2U
Block 3	Kf1S	Kf2F	Kf3N	Kf4H	Kf5S	Km1S	Km2F	Km3N	Km4H	Km5F	Cm2T	Cf1U	Cm1U	Cf2T
Block 4	Kf1F	Kf2N	Kf3H	Kf4S	Kf5F	Km1F	Km2N	Km3H	Km4S	Km5N				

Block 1, 2, 3, and 4 were presented in order. Vice versa, the order of the stimuli within each block was totally randomized.

### Experimental stimuli

Original, unmasked, version of the stimuli (NM) were retrieved by two datasets: the Karolinska Directed Emotional Faces (KDEF)^25,26^ and the Chicago Face Database (CFD)^27^. More specifically:

- *Emotion recognition*. To evaluate the effects of facemasks on the recognition of emotional expressions, we used 40 images of faces from the KDEF database. This database includes 70 (unmasked) faces depicting 7 emotional expressions from 5 different perspectives. For the current study, we selected the four following expressions: fear, sadness, joy and neutral expression – from the frontal perspective (Fig. 1A). For each of the four expressions, we selected 10 faces (5 males, 5 females) among those whose emotional recognition ratings were the highest. The selection of two negative emotions (fear and sadness) was aimed at obtaining results distinct categories of emotion sharing the same (negative) valence. In our paradigm, each selected face (N = 10) was shown 4 times (1 per facial expression) in a within subjects fashion.
- *Trust attribution and re-identification task*. Since one of the goals of our study was to investigate the effect of facemasks on perceived trustworthiness, we also included 16 images of faces from the CFD, for which a validation of the degree of trust assigned to each face is already available with an Italian sample ^67^. The CFD consists of 158 high-resolution, standardized, frontal position photographs of (unmasked) males and females between 18 and 40 years old. For the current study, we selected 16 faces (8 males, 8 females) among the most trustworthy and untrustworthy ones in a balanced fashion. Images were cropped to match the size of those from the KDEF (964 × 678 pixels). As regards the trust attribution task, we selected 8 faces (4 males and 4 females) among those who had the highest and lowest scores in trustworthiness. Each face showed a neutral expression and was shown only once during the first session.

In the re-identification task, we presented our participants with 4 CFD faces (2 males and 2 females) among those of the first session and 8 new (i.e., previously unseen) CFD faces (4 males and 4 females). In the whole design, trustworthy and untrustworthy faces were shown in a balanced manner.

All the original databases used in the current study are publicly available. To obtain visual stimuli for the masked TM and SM conditions, a professional graphic designer edited each unmasked stimulus, creating two versions of the same stimulus by superimposing two different masks: a standard medical mask and a transparent one, in which the mouth could be seen through the transparency of a plastic part (Fig. 1A).

### Measurements and Statistical analysis

- *Emotion recognition*
  The first analysis was conducted on the stimuli from the KDEF, and was aimed at verifying a difference in emotion recognition across the three conditions (NM, SM, TM)—regardless of the emotion type. For each participant, we assigned 1 point for each correctly recognized emotion and 0 points for each error. A composite score was made by averaging the sum of the 40 ratings obtained from the KDEF faces (Confidence Intervals of the median have been computed via bias corrected and accelerated bootstrap—BCa: 1000 samples). Given the violation of normal distribution assumption, we run a (non-parametric) Krusal-Wallis test with Monte-Carlo exact tests (of which we reported the significance level (p_MC_; 5000 sampled tables) as well its 99% confidence interval) considering the average scores for the three Conditions (NM, SM, TM). In case of significant effects, a Mann–Whitney tests (Bonferroni corrected) was applied as post-hoc test. Effect sizes for Mann–Whitney tests were computed using the following equation: $= \frac{Z}{\surd N}$.
  The second analysis was aimed at considering the effect of facemasks on the different emotions. We performed four Krusal-Wallis tests considering the average scores for the three Conditions (NM, SM, TM), for each emotion. Post-hoc test was conducted as in the previous analysis.
  The third analysis was aimed at considering which emotions were more affected by the presence/type of mask. For each of the three Conditions (NM, SM, TM), due to the repeated measure design, we performed a Friedman test considering the scores attributed to the four different Emotions (N, H, F, S) as dependent variables. In case of significant effects, a Wilcoxon signed-rank test was applied as post-hoc test to detect differences within different emotions.
  The direction of errors in the emotion categorization task was assessed by a chi-square test comparing the actual scores with the expected values for correct answers and for errors. Expected values for correct answers and for errors were calculated on the average value of all correct answers and errors, respectively.
- *Trust attribution*
  The first analysis was conducted on the stimuli from the CFD, and was aimed at investigating the trust attribution across the three conditions (NM, SM, TM), namely whether the presence/type of mask affects both the trust assigned to a specific face, and the consistency between (un)trustworthiness scores assigned to masked and unmasked faces. Analyses were performed on three distinct sets of data: (a) on all trust scores obtained from all stimuli of the CFD, regardless of their trust scores stored in the dataset, (b) on the trust scores obtained from the CFD stimuli validated as highly trustworthy in an Italian sample^67^ and (c) on the trust scores obtained from the CFD stimuli validated as highly untrustworthy in the same Italian sample. The difference between the scores obtained by trustworthy and untrustworthy faces was assessed by means of a Wilcoxon signed-rank test. In all the previous tests, we applied a (non-parametric) Kruskal–Wallis test considering the average scores for the three Conditions (NM, SM, TM). In case of significant effects, a Mann–Whitney tests (Bonferroni corrected) was applied as post-hoc test. Effect sizes for Mann–Whitney tests were computed as before. The same statistical procedure was conducted on the stimuli from the KDEF.
  The second analysis, also conducted on the stimuli from the KDEF, was aimed at considering which emotions were more affected by the presence/type of mask in terms of trust attribution. For each of the three Conditions (NM, SM, TM), due to the repeated measure design, we performed a Friedman test considering the scores attributed to the four different Emotions (N, H, F, S) as dependent variables. In case of significant effects, a Wilcoxon signed-rank test was applied as post-hoc test to detect differences within different emotions.
- *Re-identification task*
  Analyses were performed to investigate an effect of Condition (NM, SM, TM) on the re-identification scores with the aim of assessing the impact of the mask on the ability to remember a previously displayed face. For each participant we evaluated the percentage of correct answers by scoring each trial on a binary scale (1–0 points for each correct and incorrect responses, respectively). We ran a Kruskal–Wallis test considering the average scores for the three Conditions (NM, SM, TM). In case of significant effects, a Mann–Whitney tests (Bonferroni corrected) was applied as post-hoc test.
  Lastly, in order to control subjects and trials variability in the whole experiment, we incorporated i) subjects, ii) trials (i.e. stimuli) and iii) an additional interaction subjects*faces as random effects within three generalized linear mixed models due to the possible heterogeneity of preferences across participants and trials (GLMMs; emotion recognition; trust attribution; re-identification task) that confirmed all previous significance levels (*p* < 0.05).
